# Supplementary material for: Comprehensive Geriatric Assessment (CGA) and Optimisation Services in Older Kidney Patients: Results from the First UK-Wide Transplant Centre and Renal Unit Survey Study
Source: J Clin Med. 2025 Apr 29;14(9):3070. doi: 10.3390/jcm14093070 (PMC12073067; doi:10.3390/jcm14093070)
Supplement: Supplementary file 1 [file jcm-14-03070-s001.zip › Renal Unit Questions.pdf]

1. What is your primary clinical role?

- ◯ Consultant Nephrologist
- ◯ Consultant Transplant Surgeon
- ◯ Other (please specify)

2. Are kidney transplants performed in the unit where you work?

- ◯ Yes
- ◯ No
- ◯ I don't know

3. We know that models of care vary throughout the UK. Some renal units may perform full pre-transplant assessment prior to referral to a transplanting unit for surgical evaluation, some may perform a preliminary pre-transplant assessment (where some investigations are left to be performed at the transplanting unit), and some may not perform any part of the pre-transplant assessment.

Which of the following options best describes the pre-transplant assessment for patients over 60 with CKD Stage 5 either pre-dialysis or on dialysis at the unit where you work?

- Full pre-transplant assessment
- Preliminary pre-transplant assessment
- No pre-transplant assessment
- Other/I don't know

4. Frailty, multimorbidity, and cognitive impairment are common among patients over 60 with CKD Stage 5 either pre-dialysis or on dialysis (including potential transplant recipients). These issues can impact survival and quality of life.

Please indicate below how often each of the following is assessed in patients over 60 with CKD Stage 5 either pre-dialysis or on dialysis (including potential transplant recipients) at the unit where you work.

	Frequency of assessment
Frailty	
Multimorbidity	
Cognition	

Other/I don't know (please specify)

5. If frailty is assessed in patients over 60 with CKD Stage 5 either pre-dialysis or on dialysis (including potential transplant recipients) in the unit where you work, which validated tool is used? Please select all that apply.

- [ ] Clinical Frailty Scale (CFS)/ Rockwood
- [ ] Edmonton Frail Scale (EFS)
- [ ] Fried Frailty Phenotype
- [ ] Electronic Frailty Index (eFI)
- [ ] FRAIL Scale
- [ ] Other (please specify)/I don't know

6. If cognitive impairment/cognition is assessed in patients over 60 with CKD Stage 5 either pre-dialysis or on dialysis (including potential transplant recipients) at the unit where you work, which validated tool is used? Please select all that apply.

- [ ] Montreal Cognitive Assessment (MoCA)
- [ ] Mini Mental State Examination (MMSE)
- [ ] Addenbrooke's Cognitive Examination (ACE-III)
- [ ] Rowland Universal Dementia Assessment Scale (RUDAS)
- [ ] Other (please specify)/I don't know

7. Older patients with CKD Stage 5 can be managed with dialysis, transplantation, or supportive care. We know that frailty, multimorbidity, and cognitive impairment are common issues in this group which can impact outcomes such as survival and quality of life.

Please indicate below the extent to which you agree that each of these common issues are adequately addressed by current NHS services for patients over 60 with CKD Stage 5 managed in different ways.

	Frailty	Multimorbidity	Cognitive Impairment
Patients being considered for kidney transplant			
Patients managed with dialysis			
Patients receiving supportive care (no dialysis or plan for transplantation)			

Other/I don't know

8. Please indicate on the table below how often you discuss prognosis with patients over 60 with CKD 5 being managed in different ways.

	Frequency of prognosis discussion
Patients being considered for kidney transplant	
Patients managed with dialysis	
Patients receiving supportive care (no dialysis or plan for transplantation)	

Other (please specify)

9. Comprehensive Geriatric Assessment and optimisation (CGA) is a care process which addresses the medical, functional, and psychosocial needs of older people to develop multidimensional optimisation plans. We know from the literature that CGA improves survival and functional status in older patients.

Please indicate below the extent to which you agree that there is a role for Comprehensive Geriatric Assessment and optimisation (CGA) in patients over 60 with CKD Stage 5.

	There is a role for CGA
Patients being considered for kidney transplant	
Patients being managed with dialysis	
Patients receiving supportive care (no dialysis or plan for transplantation)	

Other/ I don't know

10. Does the unit where you work offer patients over 60 with CKD Stage 5 either pre-dialysis or on dialysis (including potential transplant recipients) access to Comprehensive Geriatric Assessment and optimisation (CGA)?

- ◯ Yes
- ◯ No
- ◯ I don't know

11. Please indicate on the table below where Comprehensive Geriatric Assessment and optimisation (CGA) is offered to patients over 60 with CKD 5 in the unit where you work. Please select all that apply.

	CGA is offered
Patients being considered for kidney transplant	☐
Patients managed with dialysis	☐
Patients receiving supportive care (no dialysis or plan for transplantation)	☐

Other/ I don't know

[ ]

12. Please indicate on the table below which professionals lead on Comprehensive Geriatric Assessment and optimisation (CGA) at the unit where you work in patients over 60 with CKD Stage 5 either pre-dialysis or on dialysis (including potential transplant recipients). Please select all that apply.

	Patients being considered for kidney transplant	Patients managed with dialysis	Patients receiving supportive care (no dialysis or plan for transplantation)
Nephrologists	☐	☐	☐
Transplant Surgeons	☐	☐	☐
Geriatricians	☐	☐	☐
Anaesthetists	☐	☐	☐
Advanced Nurse Practitioners	☐	☐	☐
Clinical Nurse Specialists	☐	☐	☐

Other (please specify)

[ ]

13. Please indicate on the table below how Comprehensive Geriatric Assessment and optimisation (CGA) is delivered to patients over 60 with CKD Stage 5 either pre-dialysis or on dialysis (including potential transplant recipients) at the unit where you work. Please select all that apply.

	Patients being considered for kidney transplant	Patients managed with dialysis	Patients receiving supportive care (no dialysis or plan for transplantation)
Outpatient Nephrology-led clinic (Please specify clinic type in comment box below)	☐	☐	☐
Outpatient Pre-Transplant Surgical Assessment clinic	☐	☐	☐
Combined Nephrology and Geriatric Medicine clinic	☐	☐	☐
Geriatric Medicine clinic	☐	☐	☐
Perioperative physicians (POPS- Perioperative medicine for Older People undergoing Surgery) clinic	☐	☐	☐

Other/Specify clinic subtype

14. At the unit where you work, who funds the substantive Comprehensive Geriatric Assessment and optimisation (CGA) service for patients over 60 with CKD Stage 5 either pre-dialysis or on dialysis (including potential transplant recipients)? Please select all that apply.

- ☐ Trust funding (please specify which directorate below)
- ☐ Specialty charity (please specify which charity below)
- ☐ Hospital charity (please specify which charity below)
- ☐ I don't know
- ☐ Other (please specify)/Specify Trust directorate

15. If the unit where you work has a pilot Comprehensive Geriatric Assessment and optimisation (CGA) service running for patients over 60 with CKD Stage 5 either pre-dialysis or on dialysis (including potential transplant recipients), who funds this? Please select all that apply.

- ☐ Trust funding (please specify which directorate below)
- ☐ Specialty charity (please specify which charity below)
- ☐ Hospital charity (please specify which charity below)
- ☐ I don't know
- ☐ Other (please specify)/Specify funding source

16. At the unit where you work, are potential transplant recipients discussed at a multidisciplinary meeting (MDM) involving Nephrologists and Transplant Surgeons?

- ◯ Yes
- ◯ No
- ◯ I don't know

17. At the unit where you work, which of the following specialties are present at the MDM where potential kidney transplant recipients are discussed? Please select all that apply.

- [ ] Nephrology
- [ ] Transplant Surgery
- [ ] Geriatric Medicine
- [ ] Anaesthetics
- [ ] Transplant Coordinators
- [ ] Nurses
- [ ] Other (please specify)

18. At the unit where you work, which of the following are discussed at the MDM for potential kidney transplant recipients? Please select all that apply.

- [ ] All potential kidney transplant recipients
- [ ] All potential living donor kidney transplant recipients
- [ ] Potential kidney transplant recipients with a failed/failing previous transplant (i.e., being worked up for second or subsequent kidney transplant)
- [ ] Potential kidney transplant recipients who are living with multimorbidity (patients with two or more chronic illnesses)
- [ ] Potential kidney transplant recipients who are older (i.e., Patients aged over 60)
- [ ] Potential kidney transplant recipients who are frail
- [ ] Potential kidney transplant recipients who lack decision-making capacity regarding transplantation
- [ ] Potential kidney transplant recipients with abnormal anatomical findings on workup (e.g., urinary outflow tract obstruction)
- [ ] Other (please specify)

19. We know that many transplant centres are interested in establishing a Comprehensive Geriatric Assessment and optimisation (CGA) service to improve outcomes for patients aged over 60 with CKD 5 (including potential kidney transplant recipients). Cost, collaboration, and clinical utility have been listed in the literature as potential barriers to this.

What do you perceive as the barriers to implementing a CGA and optimisation (CGA) service in patients over 60 with CKD Stage 5 either pre-dialysis or on dialysis (including potential transplant recipients)?

Select as many of the following options from the dropdown menu as you like. Responses are ranked from 1 to a maximum of 10, with 1 representing the greatest barrier to implementation.

	Factor
Barrier 1	
Barrier 2	
Barrier 3	
Barrier 4	
Barrier 5	
Barrier 6	
Barrier 7	
Barrier 8	
Barrier 9	
Barrier 10	

Other (please specify)

20. Please respond to this question only if the unit where you work offers a Comprehensive Geriatric Assessment and optimisation (CGA) service to patients over 60 with CKD Stage 5 either pre-dialysis or on dialysis (including potential transplant recipients).

If a CGA service is available at the unit where you work, which of the following were instrumental in establishing this service?

Select as many of the following options from the dropdown menu as you like. Responses are ranked from 1 to a maximum of 10, with 1 representing the greatest enabler to successful implementation.

	Factor
Enabler 1	
Enabler 2	
Enabler 3	
Enabler 4	
Enabler 5	
Enabler 6	
Enabler 7	
Enabler 8	
Enabler 9	
Enabler 10	

Other (please specify)

21. We know that adequate training and education are essential to the implementation of Comprehensive Geriatric Assessment and optimisation (CGA), and key determinants of its success.
Have you received adequate training or education on the use of CGA in the context of patients over 60 with CKD Stage 5 either pre-dialysis or on dialysis (including potential transplant recipients)?

- ◯ Yes
- ◯ No
- ◯ I don't need it
- ◯ I don't know/Comment

22. We know that **assessment** and **documentation** of mental capacity require a structured approach. This should include documentation of a stage one impairment of mind or brain (e.g., learning difficulties, cognitive impairment, mental health disorder). It also requires a stage two assessment of (I) understanding information, (II) retaining information, (III) weighing information (risks and benefits), (IV) communicating a decision.

We know that evaluation of mental capacity can be difficult. This can be because assessing impairment in the function of the mind or brain can be challenging and may require specialist training.

Please indicate the extent to which you agree or disagree with the following statement:

"The unit where I work has a robust method of **assessing** mental capacity for every potential kidney transplant recipient aged over 60."

- ◯ Strongly agree
- ◯ Agree
- ◯ Disagree
- ◯ Strongly disagree
- ◯ Other (please specify)

[ ]

23. Please indicate the extent to which you agree or disagree with the following statement:

"The unit where I work has a robust method of **assessing** mental capacity for every patient aged over 60 with CKD 5 who is being worked up for dialysis."

- ◯ Strongly agree
- ◯ Agree
- ◯ Disagree
- ◯ Strongly disagree
- ◯ Other (please specify)

[ ]

24. Please indicate the extent to which you agree or disagree with the following statement:

"The unit where I work has a structured approach to the **documentation** of mental capacity prior to listing a patient for kidney transplant."

Please note this does not refer to a Consent Form 4.

- Strongly agree
- Agree
- Disagree
- Strongly disagree
- Other (please specify)

25. Please indicate the extent to which you agree or disagree with the following statement:

"The unit where I work has a structured approach to the **documentation** of mental capacity for patients aged over 60 with CKD 5 who are being worked up for dialysis."

Please note this does not refer to a Consent Form 4.

- Strongly agree
- Agree
- Disagree
- Strongly disagree
- Other (please specify)

26. What incentives or support would encourage you to incorporate Comprehensive Geriatric Assessment and optimisation (CGA) more consistently in the management of patients over 60 with CKD Stage 5 either pre-dialysis or on dialysis (including potential transplant recipients)?

27. Please provide any additional comments or insights regarding the challenges, benefits, or considerations related to Comprehensive Geriatric Assessment and optimisation (CGA) in patients over 60 with CKD Stage 5 either pre-dialysis or on dialysis (including potential transplant recipients).

28. If the unit where you work currently offers or is interested in offering a Comprehensive Geriatric Assessment and optimisation (CGA) service to older kidney patients and would like to become part of a dedicated national network, please leave a contact email address below.
