# Supplementary material for: Comprehensive Geriatric Assessment (CGA) and Optimisation Services in Older Kidney Patients: Results from the First UK-Wide Transplant Centre and Renal Unit Survey Study
Source: J Clin Med. 2025 Apr 29;14(9):3070. doi: 10.3390/jcm14093070 (PMC12073067; doi:10.3390/jcm14093070)
Supplement: Supplementary file 1 [file jcm-14-03070-s001.zip › Transplant Centre Questions.pdf]

1. What is your primary clinical role?

- ◯ Consultant Nephrologist
- ◯ Consultant Transplant Surgeon
- ◯ Other (please specify)

2. Does the unit where you work undertake pre-transplant assessment in kidney patients aged over 60 with CKD Stage 5?

- ◯ Yes
- ◯ No
- ◯ Other/I don't know

3. Frailty, multimorbidity, and cognitive impairment are common among patients over 60 with CKD Stage 5 either pre-dialysis or on dialysis (including potential transplant recipients). These issues can impact survival and quality of life.

Please indicate below how often each of the following is assessed in potential kidney transplant recipients aged over 60 at the unit where you work.

	Frequency of assessment
Frailty	
Multimorbidity	
Cognition	

Other (please specify)/I don't know

4. Please indicate below the extent to which you agree that each of these common issues are adequately addressed by current NHS services for patients over 60 with CKD Stage 5 who are being considered for kidney transplant.

	Frailty	Multimorbidity	Cognitive Impairment
Patients being considered for kidney transplant			

Other/I don't know

5. Comprehensive Geriatric Assessment and optimisation (CGA) is a care process which addresses the medical, functional, and psychosocial needs of older people to develop multidimensional optimisation plans. We know from the literature that CGA improves survival and functional status in older patients.

Please indicate below the extent to which you agree that there is a role for CGA in patients over 60 with CKD Stage 5 who are being considered for kidney transplant.

- Strongly agree
- Agree
- Disagree
- Strongly disagree
- Other/ I don't know

6. Does the unit where you work offer older potential kidney transplant recipients access to a Comprehensive Geriatric Assessment and optimisation (CGA) service?

- Yes
- No
- I don't know

7. Please indicate on the table below which professionals lead on Comprehensive Geriatric Assessment and optimisation (CGA) at the unit where you work in patients aged over 60 with CKD 5 who are being considered for transplantation. Select all that apply.

- [ ] Nephrologists
- [ ] Transplant Surgeons
- [ ] Geriatricians
- [ ] Anaesthetists
- [ ] Advanced Nurse Practitioners
- [ ] Clinical Nurse Specialists
- [ ] Other (please specify)

8. Please indicate on the table below how Comprehensive Geriatric Assessment and optimisation (CGA) is delivered to patients aged over 60 with CKD Stage 5 who are being considered for transplantation at the unit where you work. Select all that apply.

- [ ] Outpatient Nephrology-led clinic (Please specify clinic type in comment box below)
- [ ] Outpatient Pre-Transplant Surgical Assessment clinic
- [ ] Combined Nephrology and Geriatric Medicine clinic
- [ ] Geriatric Medicine clinic
- [ ] Perioperative physicians (POPS- Perioperative medicine for Older People undergoing Surgery) clinic
- [ ] Other/Specify clinic subtype

9. At the unit where you work, who funds the substantive Comprehensive Geriatric Assessment and optimisation (CGA) service for potential kidney transplant recipients aged over 60?

- [ ] Trust funding (please specify which directorate below)
- [ ] Specialty charity (please specify which charity below)
- [ ] Hospital charity (please specify which charity below)
- [ ] I don't know
- [ ] Other (please specify)/Specify Trust directorate

10. If the unit where you work has a pilot Comprehensive Geriatric Assessment and optimisation (CGA) service running for potential kidney transplant recipients aged over 60, who funds this?

- [ ] Trust funding (please specify which directorate below)
- [ ] Specialty charity (please specify which charity below)
- [ ] Hospital charity (please specify which charity below)
- [ ] I don't know
- [ ] Other (please specify)/Specify funding source

11. At the unit where you work, are potential transplant recipients discussed at a multidisciplinary meeting (MDM) involving Nephrologists and Transplant Surgeons?

- ○ Yes
- ○ No
- ○ I don't know

12. At the unit where you work, which of the following specialties are present at the MDM where potential kidney transplant recipients are discussed? Please select all that apply.

- [ ] Nephrology
- [ ] Transplant Surgery
- [ ] Geriatric Medicine
- [ ] Anaesthetics
- [ ] Transplant Coordinators
- [ ] Nurses
- [ ] Other (please specify)

13. At the unit where you work, which of the following are discussed at the MDM for potential kidney transplant recipients? Please select all that apply.

- [ ] All potential kidney transplant recipients
- [ ] All potential living donor kidney transplant recipients
- [ ] Potential kidney transplant recipients with a failed/failing previous transplant (i.e., being worked up for second or subsequent kidney transplant)
- [ ] Potential kidney transplant recipients who are living with multimorbidity (patients with two or more chronic illnesses)
- [ ] Potential kidney transplant recipients who are older (i.e., Patients aged over 60)
- [ ] Potential kidney transplant recipients who are frail
- [ ] Potential kidney transplant recipients who lack decision-making capacity regarding transplantation
- [ ] Potential kidney transplant recipients with abnormal anatomical findings on workup (e.g., urinary outflow tract obstruction)
- [ ] Other (please specify)

14. We know that many transplant centres are interested in establishing a Comprehensive Geriatric Assessment and optimisation (CGA) service to improve outcomes for potential kidney transplant recipients aged over 60. Cost, collaboration, and clinical utility have been listed in the literature as potential barriers to this.

What do you perceive as the barriers to implementing a CGA service in potential kidney transplant recipients aged over 60?

Select as many of the following options from the dropdown menu as you like. Responses are ranked from 1 to a maximum of 10, with 1 representing the greatest barrier to implementation.

	Factor
Barrier 1	
Barrier 2	
Barrier 3	
Barrier 4	
Barrier 5	
Barrier 6	
Barrier 7	
Barrier 8	
Barrier 9	
Barrier 10	

Other (please specify)

15. Please respond to this question only if the unit where you work offers a Comprehensive Geriatric Assessment and optimisation (CGA) service to potential kidney transplant recipients aged over 60.

For those units with a CGA service available to them, which of the following were instrumental in establishing this service?

Select as many of the following options from the dropdown menu as you like. Responses are ranked from 1 to a maximum of 10, with 1 representing the greatest enabler to successful implementation.

	Factor
Enabler 1	
Enabler 2	
Enabler 3	
Enabler 4	
Enabler 5	
Enabler 6	
Enabler 7	
Enabler 8	
Enabler 9	
Enabler 10	

Other (please specify)

16. We know that adequate training and education is essential to the implementation of Comprehensive Geriatric Assessment and optimisation (CGA), and key determinants of its success.
Have you received adequate training or education on the use of CGA in the context of kidney transplantation for older adults?

- Yes
- No
- I don't need it
- I don't know/Comment

17. We know that **assessment** and **documentation** of mental capacity requires a structured approach. This should include documentation of a stage one impairment of mind or brain (e.g., learning difficulties, cognitive impairment, mental health disorder). It also requires a stage two assessment of (I) understanding information, (II) retaining information, (III) weighing information (risks and benefits), (IV) communicating a decision.

We know that evaluation of mental capacity can be difficult This can be because assessing impairment in the function of the mind or brain can be challenging and may require specialist training.

Please indicate the extent to which you agree or disagree with the following statement:

"The unit where I work has a robust method of **assessing** mental capacity for every potential kidney transplant recipient aged over 60."

- Strongly agree
- Agree
- Disagree
- Strongly disagree
- Other (please specify)

18. Please indicate the extent to which you agree or disagree with the following statement:

"The unit where I work has a structured approach to the **documentation** of mental capacity prior to listing a patient for kidney transplant."

Please note this does not refer to a Consent Form 4.

- Strongly agree
- Agree
- Disagree
- Strongly disagree
- Other (please specify)

19. What incentives or support would encourage you to incorporate Comprehensive Geriatric Assessment and optimisation (CGA) more consistently in the evaluation of potential kidney transplant recipients aged over 60?

20. Please provide any additional comments or insights regarding the challenges, benefits, or considerations related to Comprehensive Geriatric Assessment and optimisation (CGA) in potential kidney transplant recipients aged over 60.

21. If the unit where you work currently offers or is interested in offering a Comprehensive Geriatric Assessment and optimisation (CGA) service to older potential kidney transplant recipients and would like to become part of a dedicated national network, please leave a contact email address below.
